# Evaluating the Effects of Managed Free-Roaming Cat Populations on Prey Through Stable Isotope Analysis: A Pilot Study from British Columbia, Canada

**DOI:** 10.3390/ani15213204

**Published:** 2025-11-04

**Authors:** Valentina Martinoia, Renee Ferguson, Peter J. Wolf, Mario Carić, Mario Novak, Shelly Roche

**Affiliations:** 1 Faculty of Medicine, University of Udine, P.le Kolbe 3, 33100 Udine, Italy; 2 Faculty of Environment, Simon Fraser University, 8888 University Dr., Burnaby, BC V5A 1S6, Canada; 3 TinyKittens Society, Fort Langley, BC V1M 2S2, Canada; dr.reneeferguson@gmail.com (R.F.); shelly.roche@gmail.com (S.R.); 4 Best Friends Animal Society, 5001 Angel Canyon Road, Kanab, UT 84741, USA; peterw@bestfriends.org; 5 Center for Applied Bioanthropology, Institute for Anthropological Research, Ljudevita Gaja 32, 10000 Zagreb, Croatia; mario.caric@inantro.hr (M.C.); mario.novak@inantro.hr (M.N.); 6 Faculty of Humanities, University of Primorska, Titov trg 5, 6000 Koper, Slovenia

**Keywords:** trap-neuter-return (TNR) programs, stable isotope analysis, free-roaming cats, conservation

## Abstract

Free-roaming domestic cats (*Felis catus*) present a major management challenge for animal welfare and biodiversity conservation. Trap-neuter-return (TNR) programs, which include sterilization and return of cats, are increasingly adopted to manage cat populations, often alongside routine food provisioning. However, their effectiveness in reducing cats’ reliance on wild prey remains contested. In this study, we use stable isotope analysis (δ^13^C, δ^15^N, δ^34^S) of cat fur to investigate dietary patterns before and after TNR implementation in the context of concurrent changes in food availability linked to the closure of nearby mink-farming operations. We analyzed samples from 122 cats in a large-scale TNR initiative on a rural property in British Columbia, Canada. These included indoor cats (control), free-roaming cats prior to TNR (Group 1), a subset of Group 1 re-sampled months after food provisioning began (Run 2), and newly sampled cats that had been fed regularly before trapping (Group 2). Local prey and food sources were also analyzed to provide a comparative isotopic baseline. Our results show clear dietary shifts following TNR. Group 1 cats exhibited high isotopic variability and elevated δ^15^N and δ^34^S values, consistent with wild prey consumption. In contrast, post-TNR cats showed significantly lower and more homogeneous values, aligning closely with those of indoor, kibble-fed cats. These changes are consistent with a reduced dietary reliance on wildlife and raw mink feed following the combination of TNR with regular provisioning and the cessation of mink operations. These findings demonstrate that regular food provisioning in TNR-managed colonies, particularly when combined with broader environmental changes, can significantly alter cat diets and potentially reduce their dependence on wild prey.

## Simple Summary

Free-roaming domestic cats can affect biodiversity through their consumption of wild prey, creating challenges for both wildlife conservation and animal welfare. Trap-neuter-return (TNR) programs, which involve sterilizing and returning cats to their colonies, are increasingly used as a management approach and are often combined with regular food provisioning. However, it is uncertain whether these measures reduce reliance on prey in the diet. In this study, we investigated the diets of 122 cats on a rural property in Canada before and after the implementation of a TNR program with food provisioning and during the concurrent cessation of nearby mink-farming operations. By analyzing chemical markers in cat fur, we compared cats that lived indoors, cats before TNR, and cats after TNR with regular feeding. Before TNR, cats showed clear signs of consuming wild prey, while after TNR and food provisioning their diets shifted to resemble those of indoor cats, relying mostly on commercial food. These findings are consistent with a reduced dependence on wildlife and raw mink feed under the combined influence of TNR with food provisioning and the closure of mink operations. Our findings highlight the value of this management strategy for reducing ecological impacts of free-roaming cats while supporting humane population control.

## 1. Introduction

Trap-neuter-return (TNR) programs involve the humane trapping and sterilization of unowned, free-roaming cats (*Felis catus*). Following a brief recovery, cats are returned to where they were trapped. Many TNR programs also include vaccinations against the rabies virus and other diseases [1]. Regular feeding is an integral part of TNR efforts; it is seen as not only a fundamental caregiving component [2], but also improves trapping efficiency [3,4,5] and allows for long-term monitoring [6,7,8,9,10].

As TNR programs have become increasingly popular, one of the most common and persistent criticisms is that they fail to protect wildlife from predation [11,12]. However, although there has been some attempt to study the impact of free-roaming cats in “managed TNR colonies” [13] on wildlife, few studies have focused on the examination of the diets of such cats to provide direct evidence about their actual consumption of wild prey. Understanding this is crucial for developing public policies that balance the humane treatment of free-roaming cats with legitimate conservation concerns.

Stable isotope analysis (SIA) is a powerful tool widely used in ecological research for investigating animal diet, trophic relationships, migration, and ecological interactions [14,15,16]. It is especially valuable for free-ranging species where direct observation of feeding behavior is difficult [17], as it can also distinguish the use of anthropogenic versus natural food resources, underscoring the utility of this technique in revealing the complex interactions between free-roaming cats, their diets, and the implications for local ecosystems and informed management practices. For example, previous isotopic research conducted in the Florida Keys (U.S.) [18] utilized stable isotope analyses alongside camera traps to assess the dietary patterns of feral and free-ranging domestic cats. The study revealed that, while these cats largely relied on anthropogenic food sources, a few still exhibited significant predatory behavior. In another study on Tokunoshima Island (Japan) [19], stable isotope analysis highlighted the considerable influence of artificial resources on the feeding habits of free-ranging cats. Although some evidence of predation on native species was found, the isotopic data suggested that cats living closer to human settlements were predominantly dependent on human-provided food.

In mammals, δ^13^C values reflect the primary source of carbon, with higher values generally indicating inputs from C_4_ plants (e.g., corn or corn-fed livestock, common in pet food) [20,21], while lower values are associated with C_3_-based food webs (typical of temperate forests and native prey). δ^15^N values increase with trophic level, making them useful for assessing the proportion of animal versus plant protein in the diet [22,23]. The δ^34^S values of animal tissues reflect the sulfur isotopic composition of dietary protein, which is influenced by local soils, geology, atmospheric inputs (e.g., sea spray), and environmental factors such as rainfall and distance from the coast [24]. Terrestrial organisms typically show δ^34^S values between +5‰ and +10‰, whereas values above +14‰ in mammalian tissues often indicate a substantial contribution of marine-derived foods, such as fish or fishmeal-based pet diets [20,25]. These patterns make δ^34^S particularly useful for distinguishing terrestrial versus marine resources and for identifying processed foods containing marine ingredients or other sulfur-rich additives.

Stable isotope ratios (δ^13^C, δ^15^N, and δ^34^S), present in bodily proteins like keratin (e.g., hair/fur), provide detailed short-term records of an organism’s diet [26,27]. In the case of cat fur, these isotopic values typically reflect dietary intake on the order of roughly 1 cm of hair growth per month [28]. To our knowledge, no significant isotopic differences have been observed between surgically sterilized and non-sterilized individuals [29].

When food is assimilated into a consumer’s tissues, its isotope ratios do not transfer directly; instead, they undergo fractionation, a physiological process that results in predictable shifts in isotope values from “source” to “consumer”. This change is quantified using trophic discrimination factors (TDFs), denoted as Δ^13^C and Δ^15^N, which represent the average difference between the isotopic composition of the diet and that of the consumer’s tissue. TDFs are typically derived from controlled feeding experiments with captive animals and can vary depending on species, tissue type, diet composition, and metabolic factors [30,31]. However, published values specific to domestic cats remain scarce. While studies such as McDonald et al. [15] have estimated feline-specific TDFs (Δ^13^C = 2.6‰; Δ^15^N = 1.9‰) using a single indoor cat fed a known, consistent diet, our dataset does not allow for a direct calculation of species-specific TDFs, as we did not obtain isotopic measurements of the specific commercial food consumed by the indoor cats included in our dataset. Therefore, we adopted the general mammalian TDFs commonly used in ecological studies (Δ^13^C ≈ 1.0‰ and Δ^15^N ≈ 3.4‰) [23,30,32] as a conservative and widely accepted standard.

In our pilot study, we used δ^13^C, δ^15^N, and δ^34^S stable isotope analysis (SIA) of fur samples to investigate dietary patterns in domestic cats from British Columbia and to evaluate how these patterns shift in response to trap-neuter-return (TNR) interventions that include regular food provisioning.

## 2. Materials and Methods

### 2.1. The Study Site

The TinyKittens Society (TKS) TNR project site is located on a 22.3 ha farm in Langley. The area is characterized by a diverse landscape of barns, pastures, wooded areas, and blackberry bramble patches: features that provide natural shelter to a variety of animals, including cats, wildlife, and sheep. At the start of the TNR project, TKS identified 210 free-roaming cats on the property, with a sex distribution of 47% females and 53% males. The cats were monitored and catalogued by volunteers and attending veterinarians since January 2023, and provided consistent age estimates based on observation, dentition, and findings from physical examinations conducted prior to surgery. The age range of the cats varies: 31% are under six months old, 22.8% are between six months and two years, 26.6% are between two and five years, and 19.6% are older than five years. Post-TNR, the age range shifted significantly as no new kittens have been born on the property since October 2023.

**Figure 1:**
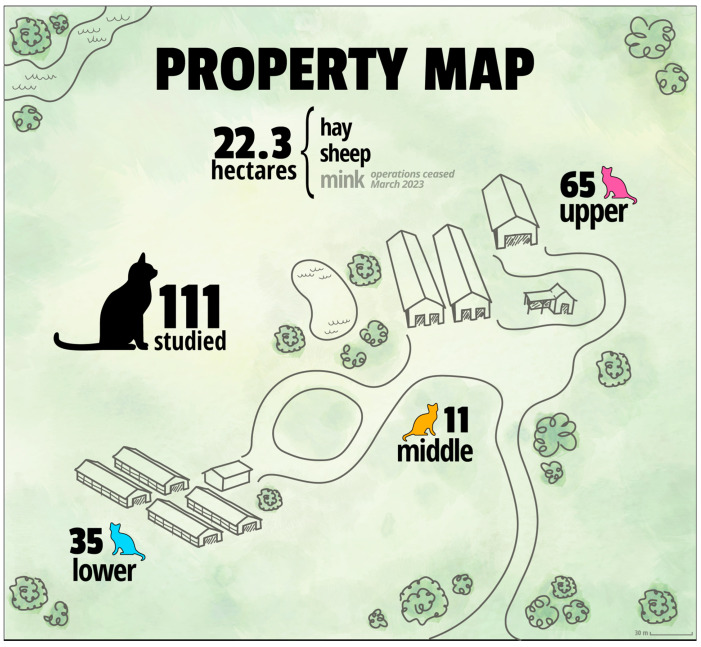
Graphic map of the TinyKittens TNR Project in Langley, British Columbia, Canada.

Historically, the property housed a mink-farming operation from the 1940s until March 2023, when all mink-related activities ceased due to British Columbia’s ban on the practice. On 9 January 2023, TKS was granted permission to implement a TNR program that included daily provisions of food for the cats and was aimed at humanely controlling the cat population, improving their health, and reducing overpopulation-related suffering. TKS identified three main groups (hereafter called “colonies”) on the farm: Upper, Middle, and Lower (Figure 1), and started monitoring the cats from each colony through daily in-person observations and trail cameras placed throughout the property.

**Figure 2:**
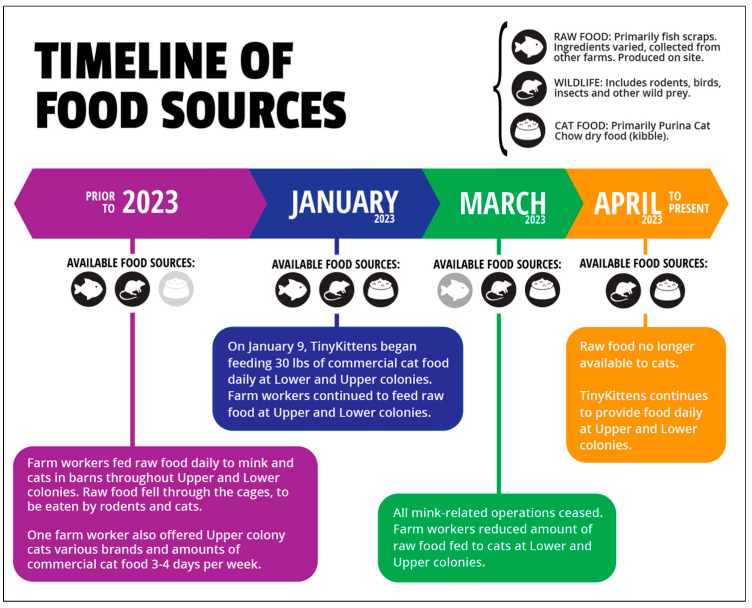
Infographic of the timeline of food sources available at the cat colony from before 2023 to the present.

The type and availability of food sources for the free-roaming cats on the farm has shifted significantly both before and throughout 2023 (Figure 2). Prior to this year, it is likely that (at least some) cats fed on some (unknown) combination of prey and raw food from the mink farm. Farm workers regularly fed raw fish scraps to the mink, much of which fell through cages or was discarded in the barns, where it attracted rodents and cats. One farm worker also sporadically provided kibble to the Upper colony cats 3–4 days a week, though the brand and quantity varied.

In January 2023, when TKS began managing the colony, they introduced a daily supply of 13.6 kg of kibble to the Upper, Middle, and Lower colonies. Farm workers continued to feed raw mink food alongside kibble until March, when mink-farming operations ceased. At that point, raw mink food feeding diminished, and by April, it stopped entirely, leaving TKS’ daily provision of kibble and (likely) prey as the only food sources available to the cats.

The TKS TNR project therefore provides an ideal context to investigate the dietary patterns of free-roaming cats through stable isotope analysis. By analyzing changes in stable isotope ratios in cat fur pre- and post-TNR and integrating this data with direct observations, the aim of this study is to gain insights into how changes in food availability and management practices influence the feeding behavior and dietary preferences of free-roaming cats.

### 2.2. Sampling

Our sampling included two key populations: (1) strictly indoor cats, reliant solely on commercial cat food (hereafter referred to as “indoor”), and (2) free-roaming cats (hereafter “free-roaming”) under the care of the TKS on a rural property in Langley, British Columbia, Canada. The study was structured in three phases to capture different stages of the TNR process. First, we analyzed fur from free-roaming cats prior to sterilization or regular provisioning (Group 1). Next, we re-sampled a subset of those cats who had by then been sterilized and had received daily kibble for at least four months, allowing us to assess dietary changes over time (Run 2). Finally, we analyzed fur from a third group (Group 2) of free-roaming cats that had been fed commercial kibble for at least five months before their trapping and sterilization. This phased approach enabled us to track dietary transitions associated with TNR and provisioning.

### 2.3. SIA—Fur and Feathers

Fur samples (140 in all) were collected from a total of 122 domestic cats in British Columbia, including both free-roaming and strictly indoor individuals. All sampling was performed under veterinary supervision during routine spay/neuter procedures, with the handling of the free-roaming cats following a low-stress procedure (https://tinykittens.com/ferals [accessed on 22 August 2025]).

Veterinarians were instructed to shave a 5 × 5 cm square from the right hindquarter and place the fur directly into labeled Ziploc^®^ bags (Varennes, QC, Canada), which were then shipped to the Institute for Anthropological Research in Zagreb (Croatia) for preparation prior to stable isotope analysis. The hindquarter region was selected as a consistently accessible and low-stress area, ensuring uniform sampling across individuals and inter-sample comparability. For isotope analysis, only the portion of fur closest to the root was used, as this reflects the most recent diet and minimizes variability associated with different hair growth periods.

The sample set includes 11 strictly indoor cats, used as a control group, all of which had been exclusively fed commercial cat food (see Supplementary Material S1). The remaining individuals (*n* = 111) were free-roaming cats managed by TKS and were divided into three groups. Group 1 includes 70 free-roaming cats sampled between January and June 2023 prior to sterilization and before the initiation of consistent feeding with commercial food. In October 2023, a second round of sampling (Run 2) was conducted on a subset of 17 cats from Group 1, who had by then been sterilized and receiving daily commercial kibble for at least four months, in order to assess isotopic changes associated with the transition to a consistent post-TNR diet. Group 2 includes 43 new fur samples from 42 cats managed by TKS: 37 adults that had been fed daily by TKS for at least five months before their sterilization procedure and fur collection, and five were kittens born in the colony and whose fur was sampled at 4–6 months of age. It is important to note that one individual (4146), originally sampled as part of Group 2, was sampled again in October 2023. This second fur sample was included in Run 2, bringing the total number of individuals in Run 2 to 18. Because this cat was not originally part of Group 1, they are introduced in the context of Run 2 only when discussing Group 2 later in the manuscript.

The fur samples were prepared for stable isotope analysis at the Institute for Anthropological Research in Zagreb following a slightly modified protocol to that described in the literature [33,34]. Specifically, variable amounts of hair strands of about 3 cm in length were placed into test tubes and rinsed 3× and then sonicated twice (10 min) in deionized water to remove macro debris. Subsequently, the samples were soaked in a solution of methanol and chloroform (2:1 *v*/*v*) for 10 min, rinsed with deionized water (3×), and soaked in 2:1 methanol:chloroform again for 2 h in order to remove contaminants such as lipids and body fluids [35,36]. The samples were then rinsed 3× with deionized water and dried into test tubes at 36 °C for 24 h in an incubation oven. The dried samples were then cut into smaller pieces (~1–3 mm), put into Eppendorf Tubes^®^ (Eppendorf, Hamburg, Germany), and shipped to Sercon Analytical in Crewe (Cheshire, UK) for δ^13^C, δ^15^N, and δ^34^S EA-IRMS analyses using a Sercon 20-20 IRMS (Sercon Limited, Crewe, UK). Detailed information about sample values, and quality controls and standards used for the analysis is presented in Supplementary Material S2.

The isotopic data for the baseline were processed and analyzed for EA-IRMS in the Stable Isotopes Laboratory, Simon Fraser University (Burnaby, Canada) using a Thermo Delta V mass spectrometer (Thermo Fisher Scientific, Waltham, MA, USA). Baseline samples were obtained from four bird feathers (two from an unidentified species and two from crows) recovered on property grounds and one rodent skeleton found in the area of the colony, as well as from the commercial (Purina^®^ Cat Chow [Nestlé Purina PetCare Company, St. Louis, MO, USA]) kibble used at the colony to feed the cats and the raw mink food that was produced on the farm. The contents of the raw mink food varied between batches, although they were generally fish-based, they could also contain poultry scraps, eggs, and/or other sources of protein. The barb of the birds’ feathers was processed for the baseline stable isotope analyses following the same protocol used for the fur.

All isotopic values are expressed in delta (δ) notation in per mil (‰) relative to international standards: VPDB (Vienna Pee Dee Belemnite) for δ^13^C, AIR (ambient inhalable reservoir) for δ^15^N, and VCDT (Vienna Canyon Diablo Troilite) for δ^34^S. Analytical precision (1σ) was assessed using repeated measurements of in-house and international quality control standards run alongside the samples (see Supplementary Material S2). For carbon and nitrogen, IA-R068 (soy protein, expected δ^13^C = −25.22‰, δ^15^N = 0.99‰) and IA-R069 (tuna protein, expected δ^13^C = −18.88‰, δ^15^N = 11.60‰) gave mean values of −25.24 ± 0.05‰ and 0.98 ± 0.04‰, and −18.88 ± 0.07‰ and 11.75 ± 0.11‰, respectively. For sulphur, IA-R061 (barium sulphate, expected δ^34^S = +20.33‰) yielded 20.35 ± 0.07‰, IA-R068 (soy protein, expected δ^34^S = +5.25‰) yielded 5.24 ± 0.12‰, IA-R069 (tuna protein, expected δ^34^S = +18.91‰) yielded 18.84 ± 0.21‰, and IAEA-SO-5 (barium sulphate, expected δ^34^S = +0.50‰) yielded 0.37 ± 0.15‰.

### 2.4. SIA—Bone

For the rodent skeleton, the bone collagen was extracted following the protocol described by Müldner and Richards [37] with the addition of an ultrafiltration step as per Brown et al. [38]. Specifically, 250–450 mg of bone was cleaned via mechanical abrasion and soaked in 0.5 M HCl at 4 °C until demineralization. Subsequently, the samples were rinsed with distilled water and gelatinized at 75 °C for 48 h in a pH 3 HCl solution. After using 9 mL Ezee filter separators (Elemtex Ltd., Gunnislake, UK) for the removal of reflux-insoluble residues, the remainder of the solution was filtered through 30-kDa MWCO ultrafilters. The supernatant of the >30-kDa fraction was frozen for 24 h and then lyophilized for stable (δ^13^C, δ^15^N, and δ^34^S) isotope analysis.

### 2.5. SIA—Cat Food

Both commercial cat food and raw mink food were processed following the protocol described in McDonald et al. [15]. Specifically, both types of food were freeze-dried for 24 h and then ground with a mortar and pestle to create a homogenous paste.

Feed samples were analyzed without lipid extraction following the method of McDonald et al. [15] in order to characterize the isotopic composition of the diets consumed. Since lipids can reduce δ^13^C and, to a lesser extent, δ^15^N values in high-fat raw tissues [39,40], we evaluated the potential magnitude of this effect using a standard C:N-based lipid-normalization model [40] as no formulas specific to commercial cat food are currently available.

### 2.6. Statistical Analysis

We applied Kruskal–Wallis tests to evaluate differences in isotopic values (δ^13^C, δ^15^N, δ^34^S) among the cat groups analyzed in this study, considering various factors such as sex, colony of origin, sterilization date, and group (i.e., indoor, Group 1, Run 2, Group 2). Indoor cats were considered a single homogeneous group without sex distinction due to their uniform isotopic signatures. This non-parametric test was chosen due to the assumption of a non-normal distribution of the data and the relatively small sample sizes. When the Kruskal–Wallis test indicated significant differences, pairwise Wilcoxon rank-sum tests with Holm correction were conducted for post hoc comparisons. In addition, for the 17 cats that were sampled both before (Group 1) and after TNR with regular food provisioning (Run 2), we performed paired Wilcoxon signed-rank tests to directly assess within-individual isotopic shifts.

Lastly, to quantify and compare isotopic niche width and overlap among the four cat groups, we applied the Stable Isotope Bayesian Ellipses in R (SIBER) framework (version 2.1.9) [41]. Standard ellipse areas (SEAs) were calculated for both δ^13^C–δ^15^N and δ^15^N–δ^34^S to represent the core isotopic niche (40% of the data), corrected for sample size and uncertainty. Pairwise niche overlap percentages were computed from the standard ellipses to assess the degree of isotopic convergence or separation among groups. These analyses provide quantitative measures of trophic diversity and dietary similarity that complement the statistical comparisons of isotopic values.

All statistical analyses were performed in R (version 4.3.1). The code used for these tests is provided in Supplementary Material S3, while the .csv files used to run the code are provided in Supplementary Material S6.

## 3. Results

### 3.1. Baseline

The isotopic results for the baseline are illustrated in Table 1. The baseline samples included commercial cat food (wet and dry), rat bone, and bird feathers (crow and unidentified species) collected on the property (Table 1). For the dry food, we analyzed two Purina^®^ Cat Chow dry kibble samples, which represent the food consistently provided to the TKS cats after TNR implementation. It should be noted that the indoor cats were not fed the same brand (see Section 3.2) and their commercial diets were not sampled directly for this study. Because δ^34^S values in pet food can vary across brands and formulations depending on the inclusion of marine-derived ingredients or other additives, some differences between the indoor cat δ^34^S values and the measured colony kibble are expected. 

The wet food sample was relatively depleted in δ^13^C (−23.8‰) and enriched in δ^15^N (7.4‰) compared to the dry food samples (δ^13^C = −19.3‰ and −18.9‰; δ^15^N = 3.3‰ and 4.2‰). Sulphur values for the commercial feeds ranged from 3.9‰ and 4.8‰ for kibbles to 7.0‰ for wet food. The atomic C:N ratios for the wet and dry food samples were relatively high (8.57, 10.82, 10.35, respectively), indicating appreciable lipid contents. As a sensitivity analysis, we applied a C:N-based lipid normalization [40], which raised the wet food δ^13^C by +5.16‰ (to −18.64‰) and the dry food δ^13^C by +7.39‰ and +6.93‰ (to −11.91‰ and −11.97‰, respectively). The corrected wet-food value remained within a plausible range, but the corrected dry-food values were unrealistically enriched. This pattern is consistent with the findings of Cecchetti et al. [42], who showed that lipid normalization models are unsuitable for commercial cat foods because of their unusually high and variable C:N ratios. Similar patterns have been documented in other felid feeding studies, where applying lipid extraction or strong normalization to prey items produced artificially low Δ^13^C values relative to consumer tissues because lipid-derived carbon contributes to keratin synthesis [17,43]. We therefore treat the C:N normalization as an upper-bound sensitivity and retain uncorrected feed δ^13^C values for the baseline context, although this limitation should be kept in mind when interpreting feed–consumer discussions throughout the study. 

The rat bone sample showed intermediate values (δ^13^C = −21.9‰, δ^15^N = 9.1‰, δ^34^S = 6.0‰). Bird feathers (crow and unidentified) showed δ^13^C values between −21.5 and −20.2‰, δ^15^N values ranging from 9.5 to 11.4‰, and δ^34^S values spanning 3.4 to 6.7‰. 

While we acknowledge that the baseline for potential wild prey is limited, it reflects the material that was available on the property at the time of sampling and was further constrained by the time and budgetary resources of the project. It is also important to note that the isotopic values of potential prey tissues (e.g., bird feathers, rat bone) were included as contextual references, but we note that tissue-specific isotopic offsets exist between keratin, bone, and other tissues, and that not all tissues are necessarily consumed by cats (e.g., feathers are often discarded or regurgitated). These baseline data therefore provide and will be used in this study only a general indication of potential prey isotopic ranges, rather than a direct quantitative match to cat fur keratin values.

### 3.2. Indoor Cats

**Figure 3:**
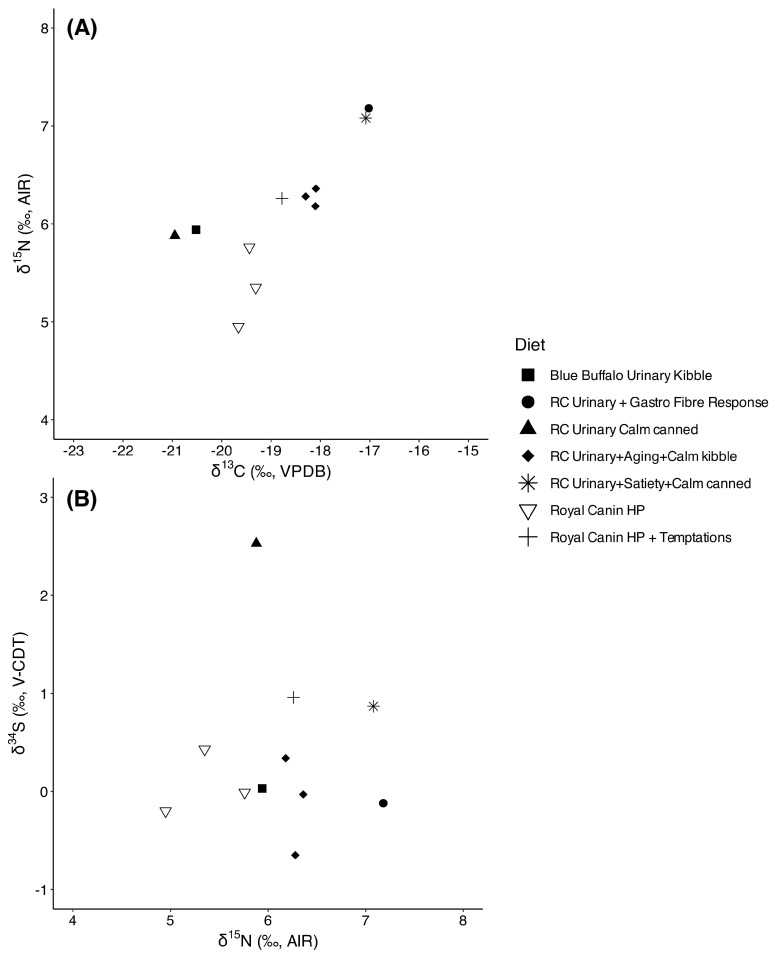
(**A**) δ^13^C (uncorrected) vs. δ^15^N and (**B**) δ^15^N vs. δ^34^S values of the indoor-only, domestic cats analyzed in this study, with indication of their diet (RC stands for Royal Canin^®^).

The stable isotope values of fur from 11 strictly indoor domestic cats, all exclusively fed commercial pet food, provide a comparative baseline for interpreting dietary variation among free-roaming individuals (Figure 3). δ^13^C values ranged from −21.0‰ to −17.0‰ for δ^13^C (δ^13^C_mean_ = −18.8 ± 1.3‰), suggesting mixed carbon inputs with variable contributions from C_4_ plant sources such as corn, which is commonly used as part of animal feed in commercial pet food formulations. δ^15^N values spanned from 5.0‰ to 7.2‰ for δ^15^N (δ^15^N_mean_ = 6.1 ± 0.7‰), reflecting the consistent consumption of foods with animal-derived protein, with differences potentially attributable to variation in protein source (e.g., poultry vs. hydrolyzed proteins), quality, or formulation. δ^34^S values were uniformly low, ranging from −0.7‰ to 2.5‰ (δ^34^S_mean_ = 0.4 ± 0.9‰) and they are consistent with terrestrial inputs and the absence of marine-derived ingredients (with the only possible exception being the cat consuming Royal Canin^®^ Urinary Calm canned [Guelph, ON, Canada]). While we lacked isotopic measurements of the specific commercial foods consumed, these indoor cats’ values represent a useful reference point for evaluating the impact of provisioning on free-roaming cats and for identifying isotopic shifts associated with access to prey or alternative food sources.

### 3.3. Free-Roaming Cats (Group 1)

The free-roaming cats from Group 1 (*n* = 70) displayed a broad range of isotopic values, consistent with a more diverse and less controlled diet. The δ^13^C values ranged from −23.2‰ to −16.7‰, with an average of −18.6 ± 0.9‰, reflecting substantial variation in carbon sources. δ^15^N values were notably higher than those observed in indoor cats, spanning 6.4‰ to 11.3‰ (mean: 9.7 ± 1.3‰), suggesting feeding at higher trophic levels or variation in protein sources. δ^34^S values also showed marked variability, ranging from −0.9‰ to 12.5‰ (δ^34^S_mean_: 7.5 ± 4.2‰), indicating differences in sulfur dietary inputs among individuals.

#### 3.3.1. Sex

**Figure 4:**
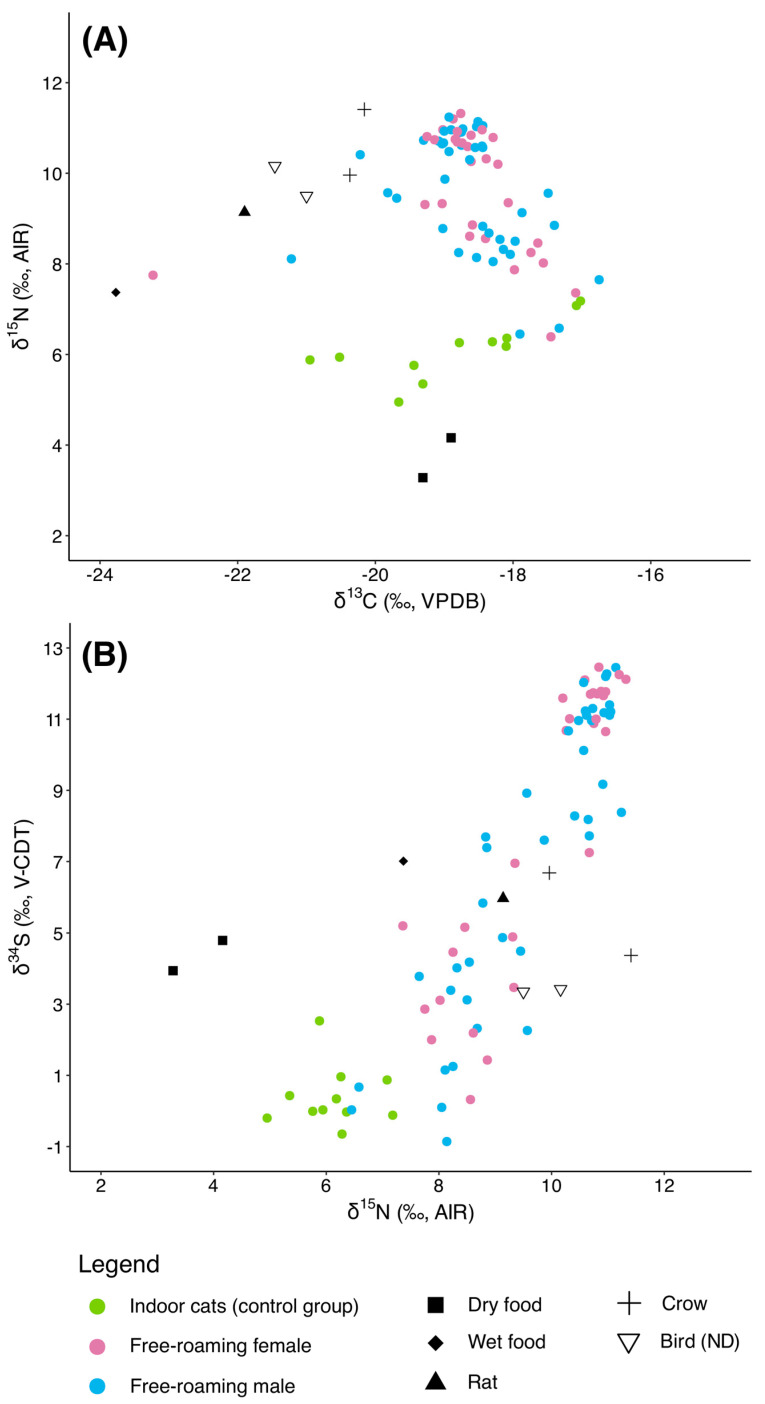
(**A**) δ^13^C vs. δ^15^N and (**B**) δ^15^N vs. δ^34^S values of the free-roaming cats analyzed in this study, divided by sex.

When analyzed by sex (Figure 4A,B), male free-roaming cats (*n* = 40) exhibited a δ^13^C mean of −18.6 ± 0.8‰ (range: −21.2‰ to −16.7‰), a δ^15^N mean of 9.6 ± 1.3‰ (range: 6.4‰ to 11.2‰), and a δ^34^S mean of 7.1 ± 4.2‰ (range: −0.9‰ to 12.5‰). Female free-roaming cats (*n* = 30) had comparable δ^13^C values (mean: −18.6 ± 1.0‰) but a wider range (−23.2‰ to −17.1‰). Their δ^15^N values averaged slightly higher than those of males at 9.7 ± 1.4‰ (range: 6.4‰ to 11.3‰), while δ^34^S values averaged 8.1 ± 4.2‰ and ranged from 0.3‰ to 12.5‰.

The Kruskal–Wallis test for δ^13^C showed no significant difference among the three groups (χ^2^ = 0.368, df = 2, *p* = 0.83), indicating consistent carbon isotope values across indoor cats, free-roaming females, and free-roaming males. In contrast, both δ^15^N and δ^34^S values differed significantly among groups, with χ^2^ values of 27.3 (df = 2, *p* = 1.15 × 10^−6^) and 24.2 (df = 2, *p* = 5.53 × 10^−6^), respectively, indicating notable variation in nitrogen and sulfur isotope signatures.

Post hoc pairwise comparisons using Wilcoxon tests revealed no significant differences in δ^13^C between any pairs of groups (all *p*-values = 1), confirming uniformity in dietary carbon sources. However, δ^15^N values for the indoor group were significantly different from those in both free-roaming females (*p* = 3.5 × 10^−6^) and free-roaming males (*p* = 2.4 × 10^−6^), while free-roaming females and males did not differ significantly from each other (*p* = 0.82). A similar pattern was observed for δ^34^S, with the indoor group differing significantly from free-ranging females (*p* = 1.7 × 10^−7^) and free-ranging males (*p* = 2.4 × 10^−5^), but no significant difference between the sexes (*p* = 0.23).

#### 3.3.2. Colonies

**Figure 5:**
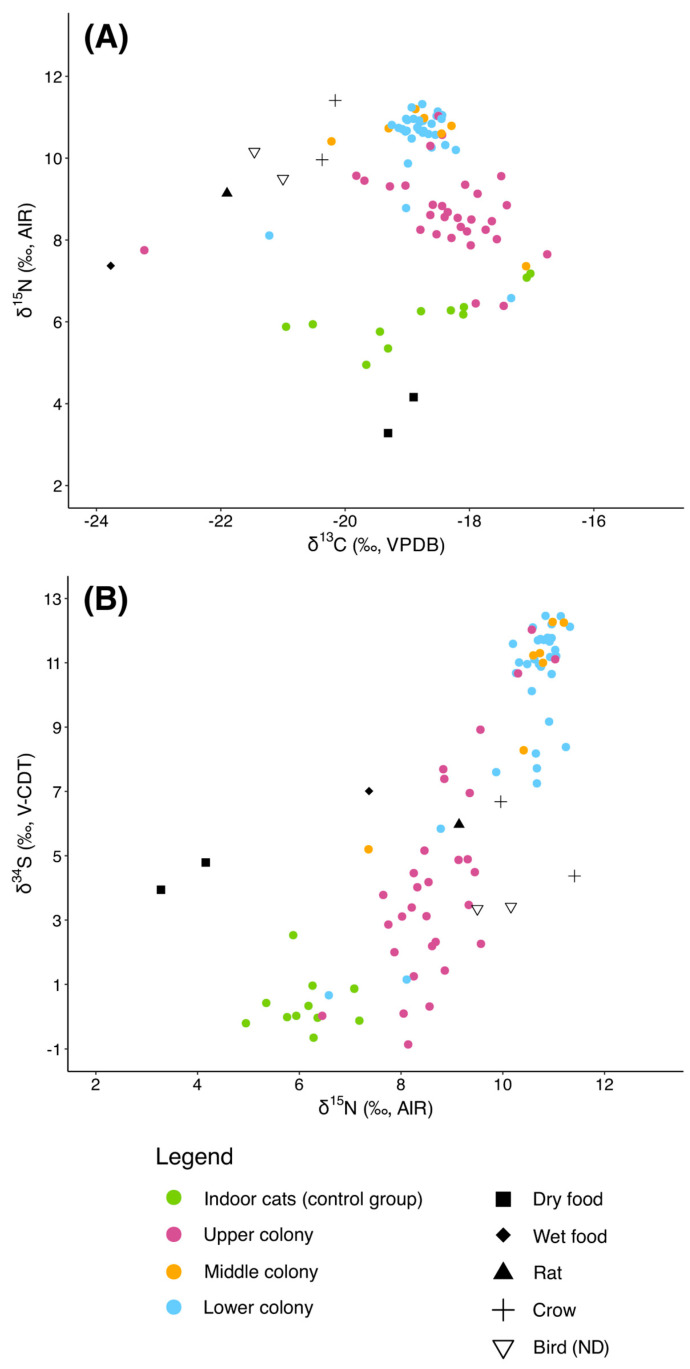
(**A**) δ^13^C vs. δ^15^N and (**B**) δ^15^N vs. δ^34^S values of the free-roaming cats analyzed in this study, divided by colony.

When analyzed by colony (Figure 5A,B), individuals from the Lower colony (*n* = 32) exhibited δ^13^C values ranging from −21.2‰ to −17.3‰, with a mean of −18.8 ± 0.6‰. Their δ^15^N values were the highest among the three colonies, averaging 10.5 ± 1.0‰ (range: 6.6‰–12.5‰). δ^34^S values were also elevated, with a mean of 10.0 ± 2.9‰ and a range of 0.7‰–13.6‰.

Cats from the Middle colony (*n* = 7) showed comparable δ^13^C values (mean: −18.7 ± 1.0‰, range: −20.2‰–17.1‰) but exhibited narrower ranges for both δ^15^N and δ^34^S compared to the Lower colony. Their δ^15^N values averaged 10.3 ± 1.3‰ (range: 7.4‰–11.2‰), while δ^34^S values averaged 10.2 ± 2.6‰ (range: 5.2‰–12.3‰), suggesting a more uniform set of dietary sources within this group. In contrast, the Upper colony cats (*n* = 31) displayed slightly lower δ^15^N values, with a mean of 8.7 ± 1.0‰ (range: 6.4‰–11.0‰). Their δ^13^C values spanned a wider interval (−23.2‰ to −16.7‰, mean: −18.4 ± 1.1‰), and δ^34^S values averaged 4.3 ± 3.3‰ (range: −0.9‰–12.0‰). These differences point to distinct dietary patterns or environmental influences compared to the other colonies.

We conducted Kruskal–Wallis tests to assess differences in isotopic values (δ^13^C, δ^15^N, δ^34^S) among the cats from three colonies (Lower, Middle, and Upper) within Group 1 and the indoor cats as well. The tests revealed significant differences for all three isotopes: δ^13^C (χ^2^ = 11.49, df = 3, *p* = 0.0094), δ^15^N (χ^2^ = 50.65, df = 3, *p* = 5.81 × 10^−11^), and δ^34^S (χ^2^ = 47.63, df = 3, *p* = 2.56 × 10^−10^), indicating substantial isotopic variability among the colonies. Subsequent pairwise Wilcoxon rank-sum tests with Holm correction were performed to identify specific group differences. For δ^13^C, significant differences were detected only between the Upper colony and the Middle colony (*p* = 0.002), while all other pairwise comparisons were not significant (*p* ≥ 0.846). Regarding δ^15^N, most pairwise comparisons showed significant differences: the indoor cats group differed significantly from the Lower (*p* = 6.8 × 10^−6^), Middle (*p* = 0.00019), and Upper colonies (*p* = 8.2 × 10^−6^), while the Lower and Middle colonies did not differ significantly (*p* = 0.927). The Upper colony also differed significantly from both the Lower (*p* = 3.0 × 10^−7^) and Middle (*p* = 0.012) colonies. For δ^34^S, a similar pattern was observed: the indoor cats group differed significantly from the Lower (*p* = 9.0 × 10^−6^), Middle (*p* = 0.00025), and Upper (*p* = 0.00031) colonies; the Upper colony also differed significantly from Lower (*p* = 1.4 × 10^−6^) and Middle (*p* = 0.00031), while the Lower and Middle colonies did not differ significantly (*p* = 0.728) between themselves. These results are summarized in Supplementary Material S5 (Table S5), highlight distinct isotopic signatures among colonies, suggesting spatial or ecological variability in diet or habitat use within Group 1.

#### 3.3.3. Sterilization Date

For completeness, we conducted additional statistical analyses to investigate potential differences in isotopic dietary signatures (δ^13^C, δ^15^N, and δ^34^S) among cats from Group 1 according to their sterilization dates, which spanned from January to June 2023. Due to the large number of monthly subgroups and complexity of results, these analyses are not discussed in detail in the main text but can be viewed in full in the Supplementary Material S1. However, scatterplots illustrating the distribution of isotopic values relative to sterilization dates are provided in Supplementary Material S4.

We first applied Kruskal–Wallis tests, which indicated significant differences for δ^13^C (*p* = 0.038), δ^15^N (*p* = 0.022), and δ^34^S (*p* = 0.012). Subsequently, pairwise Wilcoxon rank-sum tests with Holm *p*-value adjustment were performed to identify specific-group differences. For δ^13^C, significant differences were found between cats sterilized in January versus April 2023 (adjusted *p* = 0.026), with other comparisons showing no significant differences after correction. For δ^15^N, significant differences were observed between January and February 2023 (adjusted *p* = 0.003), as well as between January and March (*p* = 0.025) and January and April (*p* = 0.049); no differences were detected among the other pairs. For δ^34^S, after excluding individuals with missing sulfur values, significant differences emerged between January and February (adjusted *p* = 0.001), and January and March (*p* = 0.003), while the other comparisons were not significant. It should be noted that the Wilcoxon tests generated warnings regarding tied values, which prevented exact *p*-value calculations; however, the normal approximations used remain valid.

### 3.4. Run 2

**Figure 6:**
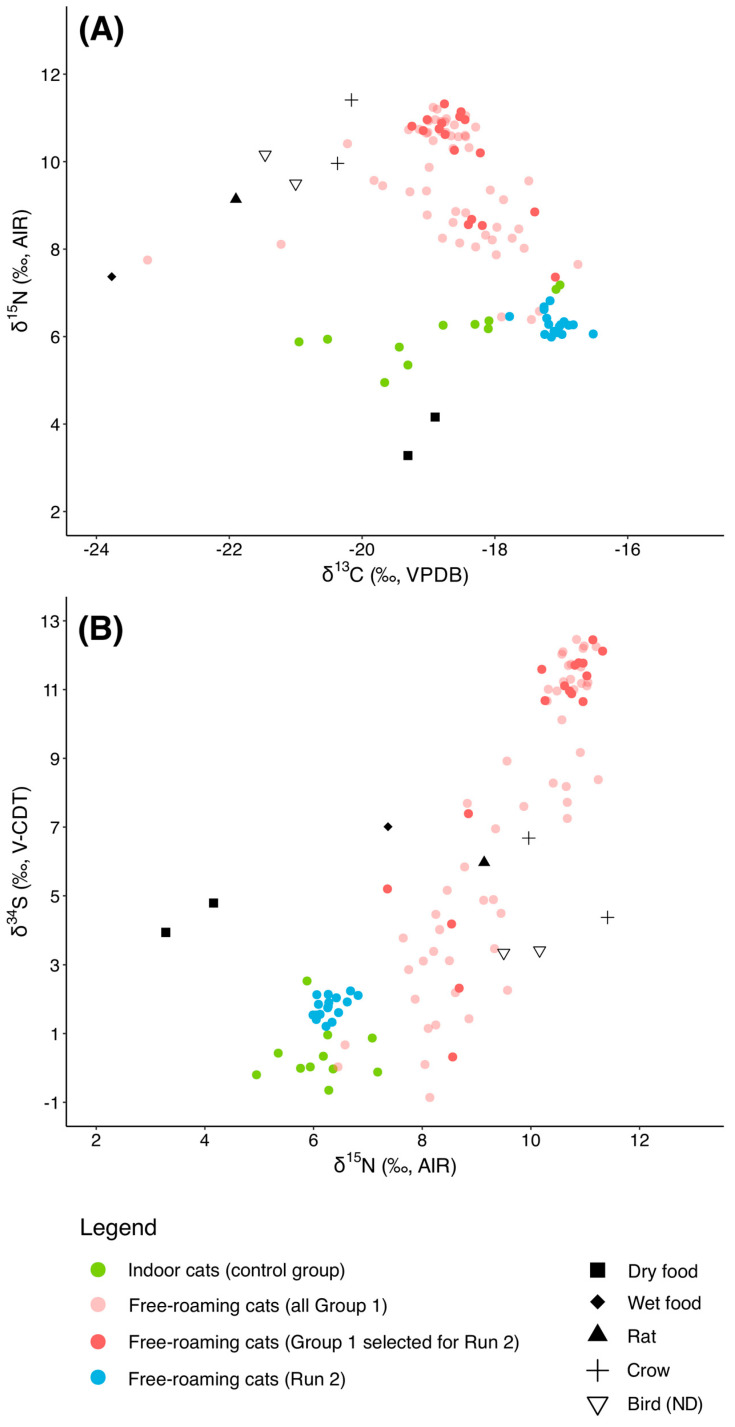
(**A**) δ^13^C vs. δ^15^N and (**B**) δ^15^N vs. δ^34^S values of the individuals from Group 1 (those selected for a re-run are in dark pink) and for individuals from Run 2.

As the second phase of the project, in October 2023, we re-sampled and analyzed fur from 17 cats who, by that time, had been sterilized and had access to TKS’ daily provision of kibble for at least four months (Run 2). We compared these follow-up data to the pre-TNR results for the same individuals to assess potential changes in dietary patterns. Run 2 comprised 7 male and 10 female cats, including 12 from the Lower colony, four from the Upper colony, and one from the Middle colony. All samples for this re-analysis were selected using standardized random selection procedures. The results of phase 2 (Figure 6) show a significant dietary shift from pre-TNR (Group 1) to post-TNR (Run 2).

Prior to TNR, the average δ^13^C values for the 17 cats were −18.5 ± 0.6‰ while after TNR, they show an offset of 1.4‰, with an average δ^13^C value of −17.1 ± 0.3‰. The δ^15^N values show an offset of 3.8‰ between Group 1 (average δ^15^N value: 10.1 ± 1.2‰) and Run 2 (average δ^15^N value: 6.3 ± 0.2‰). Lastly, δ^34^S shows the biggest offset between the two runs (7.4‰), with values going from 9.2 ± 3.8‰ in Run 1 to 1.8 ± 0.3‰ in Run 2.

Therefore, in Run 2, the isotopic values of the 17 re-analyzed cats align closely with those of the indoor cats control group, especially for δ^15^N and δ^34^S.

**Figure 7:**
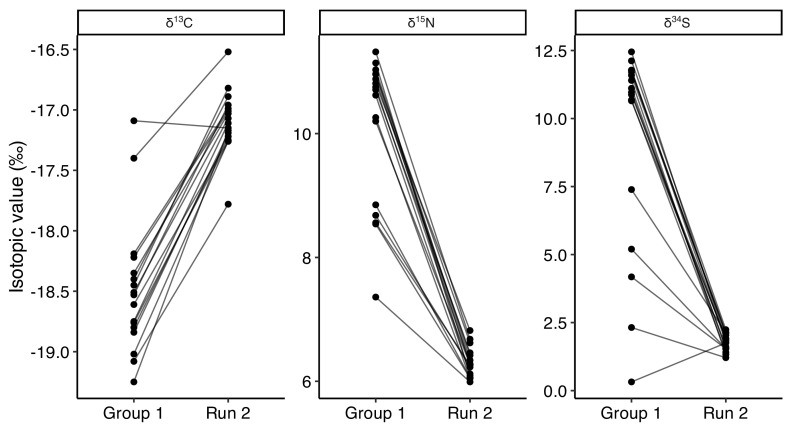
Paired isotopic values (δ^13^C, δ^15^N, δ^34^S) for 17 cats sampled before (Group 1) and after (Run 2) TNR implementation with regular food provisioning and concurrent closure of mink farm operations.

Paired Wilcoxon signed-rank tests for the 17 re-sampled cats (Group 1 vs. Run 2) confirmed significant within-individual dietary shifts. δ^13^C values increased (HL shift = +1.45‰, 95% CI +1.26 to +1.60‰, V = 152, *p* < 0.001, r = 0.86) while both δ^15^N and δ^34^S values markedly decreased (δ^15^N: HL shift = −3.96‰, 95% CI −4.47 to −3.28‰, V = 0, *p* < 0.001, r = −0.87; δ^34^S: HL shift = −8.85‰, 95% CI −9.66 to −5.43‰, V = 2, *p* < 0.001, r = −0.85). Effect sizes were uniformly large, and the rank-biserial correlations (|r| > 0.95) indicate nearly all cats shifted consistently in the same direction. These results strengthen the between-group comparisons and demonstrate a clear individual-level transition from prey and raw mink feed toward commercial food following TNR and regular provisioning (Figure 7).

### 3.5. Group 2

For the third phase of the project, we analyzed 43 new samples from free-roaming cats managed by TKS. Fur samples for all these cats were collected between June 2023 and October 2024. It must be noted that one individual (4146) was sampled twice: the first fur sample, collected in June 2023, is included in Group 2, while a second sample, collected in October 2023 (four months after sterilization and ten months after the onset of regular feeding) is included in Run 2 but is only introduced at this point in the paper, as the earlier discussion of Run 2 results focused on individuals originally from Group 1. Notably, the isotopic values of this individual show minimal changes between the two time points (Group 2: δ^13^C = −17.2‰, δ^15^N = 6.3‰, δ^34^S = 1.9‰; Run 2: δ^13^C = −17.06‰, δ^15^N = 6.04‰, δ^34^S = 2.91‰), indicating that the cat was already consuming a commercial-food-based diet at the time of the first sampling.

**Figure 8:**
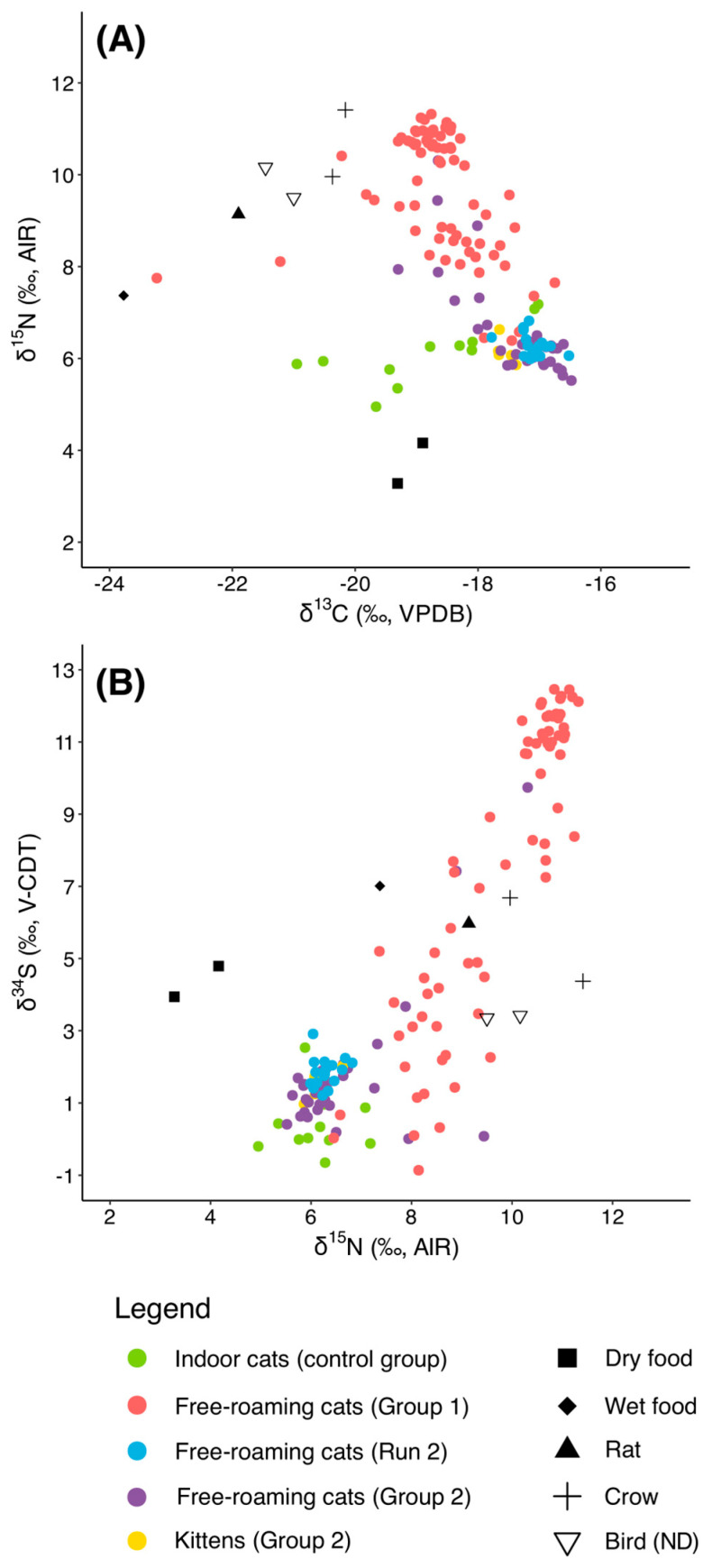
(**A**) δ^13^C vs. δ^15^N and (**B**) δ^15^N vs. δ^34^S values of the individuals from Group 2 compared with those from the control group, Group 1, and Run 2.

Our results (Figure 8) show that the cats from Group 2 exhibited isotopic values markedly different from those of Group 1, with an average δ^13^C value of −17.4 ± 0.7‰, a mean δ^15^N value of 6.5 ± 1.0‰, and an average δ^34^S value of 1.7 ± 1.7‰. These values (particularly δ^15^N and δ^34^S) closely align with those of the indoor control group rather than the highly variable isotopic signatures of the free-roaming cats in Group 1.

Notably, five individuals in Group 2 (4106, 4125, 4141, 4142, and 4144) displayed δ^15^N values substantially higher than the group mean (all > 7.5‰). Among these, only two cats (4106 and 4144) also exhibited δ^34^S values exceeding 7‰, whereas the mean δ^34^S value for Group 2, excluding these two outliers, was 3.7‰.

The five nursing kittens in Group 2 (4109, 4110, 4114, 4115, and 4116) did not exhibit the elevated isotopic values typically expected in nursing individuals. This may be because the fur analyzed had begun growing in utero or shortly after birth and therefore still reflected the isotopic composition of their mothers’ diet rather than the enriched values associated with nursing. Importantly, their values fell within the broader Group 2 range and did not skew group-level patterns or conclusions.

**Figure 9:**
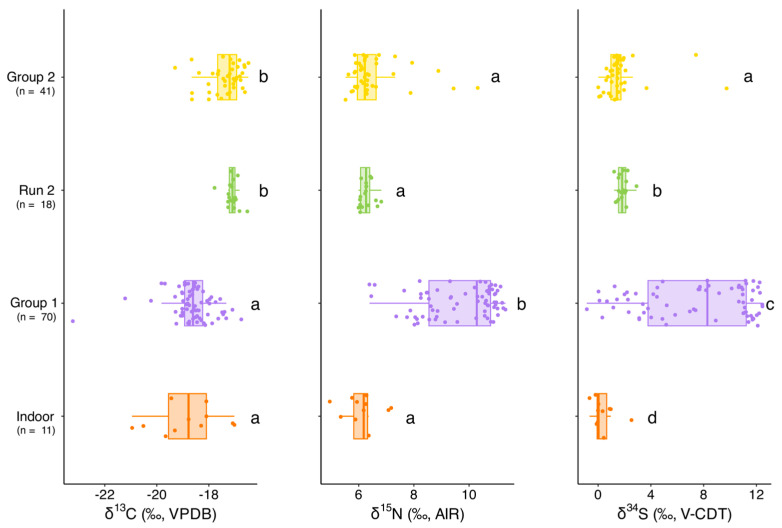
Boxplots showing δ^13^C, δ^15^N, and δ^34^S values for indoor cats, Group 1 (pre-TNR), Run 2 (re-sampled post-TNR), and Group 2 (provisioned prior to capture). Boxes indicate the interquartile range (25th–75th percentiles), whiskers extend to 1.5× the interquartile range, the horizontal line marks the median, and all points are shown with jitter. Statistical comparisons (pairwise Wilcoxon rank-sum tests with Holm correction) revealed highly significant differences between Group 1 and both Run 2 and Group 2 across all isotopes (*p* < 10^−9^), indicating strong dietary shifts after TNR and provisioning. In contrast, Run 2 and Group 2 did not differ significantly (δ^13^C: *p* = 0.40; δ^15^N: *p* = 0.92; δ^34^S: *p* = 0.004). Paired Wilcoxon signed-rank tests for re-sampled cats (Run 2) confirmed these within-individual shifts with large effect sizes (|r| ≥ 0.85). Different letters above the boxes indicate significant differences between groups based on pairwise Wilcoxon rank-sum tests with Holm correction (*p* < 0.05).

We conducted Kruskal–Wallis tests and post hoc Pairwise Wilcoxon tests to investigate differences between the four groups of cats (indoor, Group 1, Run 2, and Group 2). The results of the statistical analyses are summarized in Supplementary Material S5 (Table S5). The Kruskal–Wallis tests revealed highly significant differences among the four groups (Control, Group 1, Run 2, and Group 2) for all three isotopes (δ^13^C, δ^15^N, and δ^34^S; *p* < 0.0001 for all). Pairwise Wilcoxon comparisons showed that Group 1 (pre-TNR) was consistently distinct from the other groups. For δ^13^C, Group 1 differed significantly from both Group 2 and Run 2, whereas Group 2 and Run 2 were statistically similar, suggesting that following TNR and consistent feeding, δ^13^C values shifted to a new, stable range. The control group showed δ^13^C values comparable to Group 1 but significantly different from Group 2 and Run 2. Similarly, δ^15^N values in Group 1 were significantly higher than those observed in the other groups, while Group 2, Run 2, and the control group were statistically indistinguishable, further confirming a dietary shift post-TNR towards values consistent with indoor cats. In contrast, δ^34^S values displayed a more complex pattern: almost all pairwise comparisons, including those between Group 2 and Run 2, were significant. This greater variability in δ^34^S may reflect additional ecological or prey-source influences beyond the dietary changes captured by δ^13^C and δ^15^N (Figure 9).

**Figure 10:**
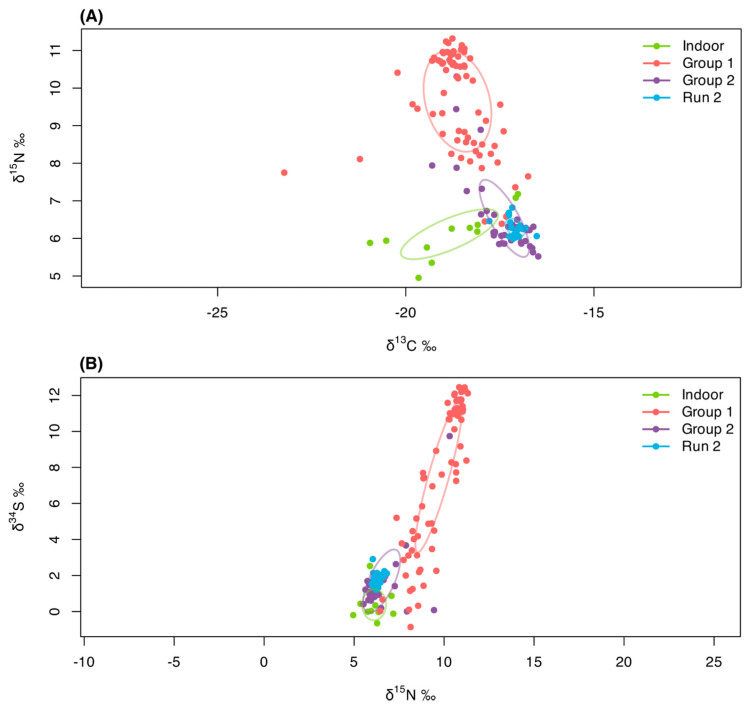
(**A**) δ^13^C vs. δ^15^N and (**B**) δ^15^N vs. δ^34^S biplots showing the isotopic niches of the four cat groups. Points represent individual isotope values, and solid ellipses indicate the 40% standard ellipse areas (SIBER). The markedly smaller and fully overlapping ellipse of Run 2 with Group 2 illustrates dietary convergence following TNR.

To further quantify isotopic niche width and overlap between groups, we applied the SIBER framework [41]. The standard ellipse areas (SEAs) confirmed the contraction of isotopic niche width following TNR in both isotopic systems (Figure 10).

In the δ^13^C–δ^15^N space, Group 1 displayed the broadest niche (SEA = 3.62‰^2^), followed by Indoor (1.76‰^2^) and Group 2 (1.33‰^2^), while Run 2 showed an extremely narrow niche (0.17‰^2^). A similar pattern was observed in the δ^15^N–δ^34^S space, with Group 1 again showing the widest niche (7.62‰^2^), followed by Group 2 (4.10‰^2^), Indoor (1.77‰^2^), and Run 2 (0.30‰^2^). Pairwise ellipse overlap revealed complete isotopic convergence (100%) between Group 2 and Run 2 in both biplots, whereas Group 1 did not overlap with any other group, confirming its distinct prey-based diet. A moderate overlap (~7% in δ^13^C–δ^15^N and ~64% in δ^15^N–δ^34^S) between Indoor and Group 2 cats indicates partial similarity in the consumption of food sources.

## 4. Discussion

This study provides robust isotopic evidence that trap-neuter-return (TNR) programs incorporating regular food provisioning can significantly alter the dietary profiles of free-roaming domestic cats. By combining stable isotope analyses of carbon (δ^13^C), nitrogen (δ^15^N), and sulfur (δ^34^S) with a phased sampling design, we evaluated the dietary transitions of cats from a former mink farm in British Columbia before and after enrollment in the TKS TNR program. Our results confirm that dietary composition markedly shifted across the study period, although these changes likely reflect the combined effects of both consistent provisioning and the concurrent closure of mink operations. Importantly, the data also highlight individual and spatial variability in foraging behavior and trophic positioning within the cat colonies.

### 4.1. Dietary Differences Between Indoor and Free-Roaming Cats

This study explored how the dietary patterns of free-roaming domestic cats are shaped by TNR programs, with a specific focus on the effects of consistent food provisioning. Using δ^13^C, δ^15^N, and δ^34^S values from fur samples, we captured dietary variability across different groups and timeframes, allowing us to track changes in individual and group-level feeding behaviors over the course of the TKS TNR initiative. The results demonstrate a clear isotopic distinction between pre- and post-TNR free-roaming cats, with post-TNR values closely resembling those of strictly indoor, commercially fed cats. These findings reinforce the idea that consistent provisioning of commercial cat food (in our case, together with the cessation of mink farming) can significantly reduce cats’ dietary reliance on wild prey, thereby lessening their potential ecological impact.

### 4.2. Pre-TNR Variability: Group 1

The high variability in isotopic values observed in Group 1 reflects a diverse range of dietary inputs among free-roaming cats prior to TNR. Elevated δ^15^N values (significantly higher than those of indoor cats) observed among this group (particularly for the individuals from the Lower colony) suggest that these individuals consumed diets rich in animal protein, sourced from a combination of raw mink food waste and, possibly, wild prey. This interpretation is supported by our baseline measurements: rats and wild birds on the property displayed δ^15^N values between ~9‰ and 11.4‰, values broadly overlapping with those of Group 1 cats. δ^34^S values in Group 1 also ranged widely, from highly terrestrial (~0‰) to values above 12‰, consistent with inputs from marine-rich sources such as the fish-based mink feed from the nearby mink farm, which itself averaged δ^34^S values around 7.0‰. Statistical comparisons between Group 1 and all other groups (Run 2, Group 2, indoor) confirmed highly significant differences across all isotopic dimensions, particularly for δ^15^N (*p* = 7.0 × 10^−15^) and δ^34^S (*p* = 1.3 × 10^−10^). This supports the interpretation that Group 1 cats accessed a range of heterogeneous food sources before TNR, including prey and anthropogenic waste.

Sex-based comparisons within Group 1 did not reveal significant isotopic differences, suggesting that males and females had similar access to dietary resources and feeding behaviors. However, notable dietary variation was observed among colonies, with cats from the Lower and Middle colonies exhibiting significantly higher δ^15^N and δ^34^S values compared to the Upper colony (*p* < 0.01). These differences likely reflect spatial variability in access to prey and mink feed prior to the cessation of mink operations in April 2023. In particular, the Lower colony, which was located near the mink barns, appears to have had the greatest access to raw food waste.

Interestingly, some variation in isotopic values within Group 1 also tracked with sterilization month. Cats sterilized earlier in the year (especially January and February) exhibited significantly higher δ^15^N and δ^34^S values than those sterilized in later months (April–June), which may reflect seasonal or logistical shifts in food availability (e.g., decreased access to mink waste after April).

### 4.3. Dietary Transitions Post-TNR: Run 2 and Group 2

Cats re-sampled several months after TNR and consistent feeding (Run 2) displayed isotopic profiles strongly resembling those of indoor, commercially fed cats, particularly in δ^15^N and δ^34^S. This suggests a substantial reduction in the consumption of prey and mink feed, and near-exclusive reliance on kibble post-TNR. The δ^13^C values in Run 2 also shifted significantly (1.4‰ increase), indicating increased input from C_4_-derived ingredients (e.g., corn), common in commercial pet food formulations. Although δ^13^C values are less trophically informative, they reinforce the overall dietary transition and are consistent with our baseline values for kibble (−19‰).

Two major factors likely contributed to this transition. First, the closure of the mink farm in April 2023, which had provided raw food and fish scraps to the cats, removed a significant food source. With the mink farm no longer operating, the cats had to rely more heavily on the food provided by TKS. Notably, while cats from the Lower colony had previously relied on mink farm waste, it is expected that they switched to kibble after the farm’s closure. However, the fact that cats from the Upper colony, whose pre-TNR diet showed evidence of prey consumption, also demonstrated altered isotopic values after TNR, suggests that over the long term, TNR may contribute to dietary shifts away from prey, although the present methods capture diet rather than hunting behavior, and further research is needed to assess the latter. The second factor is the daily availability of kibble at the colony starting in January 2023. This new food source likely prompted the cats to opportunistically rely on it rather than actively consuming wild prey or scavenging. This is consistent with previous research showing that regularly fed cats are less reliant on wild prey [18,44].

Group 2 cats, who had been consistently fed prior to sampling, showed remarkably similar isotopic profiles to those of Run 2, with no significant difference in δ^13^C (*p* = 0.4039) and δ^15^N (*p* = 0.92). These patterns suggest that free-roaming cats, even if not yet sterilized, but regularly provisioned, undergo similar dietary shifts away from prey or waste food sources. However, five individuals in Group 2 presented elevated δ^15^N values (>7.5‰), with two also showing high δ^34^S values (>7‰). These outliers may represent recent dietary deviations (e.g., active consumption of prey or scavenging) or longer-term isotopic carryover due to fur turnover rates. Since fur reflects dietary intake over previous weeks or months, such values may indicate residual feeding on prey or fish-based waste shortly before or during the transition to a kibble-based diet. The presence of these outliers highlights the importance of considering individual behavioral variability within managed colonies. In contrast, the five nursing kittens in Group 2 did not show isotopic enrichment typically associated with nursing, which may be due to the timing of fur growth. If the analyzed fur formed in utero or shortly after birth, its isotopic composition would reflect the maternal diet rather than lactation-related enrichment. This reinforces the need for caution when interpreting isotope data from juveniles.

The patterns revealed by the SIBER isotopic niche analysis reinforce our interpretations. When visualized as standard ellipses, the isotopic niches of the groups illustrate a clear contraction of dietary space after TNR and regular feeding. Before TNR, Group 1 occupied a wide and variable isotopic niche (SEA = 3.62‰^2^ in δ^13^C–δ^15^N and 7.62‰^2^ in δ^15^N–δ^34^S), consistent with a diet derived from diverse prey and waste sources. By contrast, cats that were regularly provisioned (both those captured before sterilization [Group 2] and those from Group 1 re-sampled after TNR [Run 2]) occupied much narrower and largely overlapping niches (for δ^13^C–δ^15^N and δ^15^N–δ^34^S: Group 2: 1.33‰^2^ and 4.10‰^2^; Run 2: 0.17‰^2^ and 0.30‰^2^), reflecting a shift toward homogeneous, kibble-based diets. The complete overlap between Group 2 and Run 2 ellipses (100%) highlights that dietary change occurred rapidly once consistent food provisioning was established, regardless of sterilization status. The moderate overlap between Group 2 and indoor cats (~7% in δ^13^C–δ^15^N and ~64% in δ^15^N–δ^34^S) further suggests that these free-roaming but managed individuals were already relying heavily on the same anthropogenic food resources as indoor pets. In ecological terms, the progressive narrowing of isotopic niche space captures a loss of trophic diversity and a growing dependence on human-provided resources: a predictable but quantitatively demonstrable outcome of TNR programs combined with sustained feeding.

### 4.4. Broader Implications and Ecological Relevance

The alignment between post-TNR isotopic values and those of indoor cats provides compelling evidence that consistent food provisioning can reduce the consumption of wild prey/scavenging by free-roaming cats, especially when implemented early and uniformly. These findings support earlier camera-based studies showing reduced hunting behavior in managed colonies [13] and align with stable isotope studies conducted in other contexts.

For example, Cove and colleagues [18] examined feral cats from the Florida Keys and observed broad δ^13^C and δ^15^N ranges indicative of mixed consumption of anthropogenic and wild prey resources. Cats captured away from human settlements on Key Largo, and some individuals from Big Pine Key, derived over 50% of their diet from wild prey. In contrast, our post-TNR cats (Groups 2 and Run 2) exhibited much narrower isotopic niches (SEA_40_ = 1.33 and 0.17‰^2^, respectively), reflecting a reduced dietary breadth and greater dependence on provisioned foods.

Similarly, on Tokunoshima Island (Japan), Maeda et al. [19] combined fecal and isotopic analyses to show that both “feral” (independent from humans) and “stray” (partially dependent) cats relied predominantly on human-derived food, even when trapped in forested areas. Their results suggested frequent movement between villages and natural habitats and emphasized that consistent feeding sustains large free-roaming populations. Our results also indicate that provisioning is the main driver of dietary stabilization in managed colonies. However, unlike the Tokunoshima cats, which continued to access both natural and artificial resources to some degree, the free-roaming cats in our study showed a much sharper and nearly complete transition to kibble-based diets.

Comparable conclusions were drawn by Cecchetti et al. [42], who, using behavioral and stable isotope data from owned cats in southwestern England, found that although many individuals regularly hunted and killed wild animals, their isotopic signatures reflected a predominant reliance on commercial pet food, with wild prey contributing only marginally to their overall diet. Likewise, our results show that, despite access to outdoor environments, free-roaming cats depend almost entirely on commercial cat food once it is provided consistently. Although some individuals may continue to hunt, their consumption of wild prey appears minimal and likely opportunistic rather than nutritionally significant.

Our findings underscore the importance of regular provisioning not only as a welfare measure but also as a potential conservation tool. In the TK context, consistent feeding emerges as the primary factor redirecting free-roaming cats away from foraging behaviors that could be ecologically disruptive, fostering instead a dependence on predictable, human-supplied resources.

It is also worth noting that δ^34^S showed the most persistent variability across groups. While Run 2 and Group 2 values were substantially lower than those of Group 1, some overlap remained. This variability may reflect residual marine-derived additives in commercial cat food or spatial heterogeneity in local sulfur baselines (e.g., soil or sea spray inputs). Importantly, few previous isotopic studies of free-roaming cats have included sulfur analyses, yet in our dataset δ^34^S proved particularly informative in distinguishing pre- and post-TNR dietary patterns. The inclusion of this isotope highlights subtle differences in protein source and environmental context that are not always captured by δ^13^C and δ^15^N alone, further reinforcing the evidence for a broad dietary shift following TNR and regular provisioning.

### 4.5. Limitations and Future Directions

One limitation of this study is the lack of isotopic data for all food sources consumed prior to TNR, especially prey and waste food from mink operations. While our baseline measurements are informative, they are limited, and a more comprehensive isotopic map of local rodents, birds, and mink feed batches would allow for finer-grained dietary reconstructions. Future work could also combine isotope analysis with behavioral tracking or fecal DNA analysis to corroborate prey consumption patterns.

Another limitation of our dataset is the absence of one δ^34^S value (sample 3834, Group 1). This was due to technical reasons (specifically, no material remained after C/N analysis) and not because of issues of data quality. However, the δ^13^C and δ^15^N values from this individual were broadly consistent with those of other Group 1 cats, indicating that its exclusion does not bias the group-level or temporal comparisons.

A further consideration concerns the baseline cat food samples. While cat fur was lipid-extracted prior to analysis, the commercial feeds we analyzed (provided to free-roaming cats) were not. Because lipids can lower δ^13^C, this mismatch could introduce a small systematic offset in baseline–consumer comparisons. Moreover, we did not have isotopic measurements for the specific foods consumed by the indoor control cats. Although these issues do not change the overall interpretation of strong dietary differences between groups, they should be noted as methodological limitations.

Moreover, while we adopted general mammalian TDFs rather than feline-specific values, we note that our qualitative conclusions are robust to reasonable variation in TDF estimates, given the magnitude of the isotopic differences observed between groups.

Lastly, while this study shows clear dietary transitions, it cannot address whether these shifts will persist over longer timeframes (e.g., >1 year), nor whether cats may opportunistically hunt or forage even when regularly fed. Longitudinal studies that track individuals over multiple years could provide further insight into these dynamics.

## 5. Conclusions

This study contributes important new evidence to the ongoing debate surrounding the ecological impact of free-roaming cats and the efficacy of trap-neuter-return (TNR) programs. By employing stable isotope analysis of carbon (δ^13^C), nitrogen (δ^15^N), and sulfur (δ^34^S) in cat fur, we were able to reconstruct dietary profiles across different stages of TNR implementation, capturing both individual- and population-level responses to consistent food provisioning. Our findings reveal a marked isotopic transition from highly variable, prey-influenced diets in pre-TNR cats to more homogenous profiles in post-TNR cats that closely resemble those of strictly indoor, commercially fed individuals. Our findings have important implications for both animal welfare and conservation policy. They support the position that TNR, when accompanied by systematic feeding and monitoring, can mitigate one of the most persistent criticisms of free-roaming cats: their impact on wildlife through the consumption of wild prey. By shifting dietary reliance away from prey species and toward controlled, human-provided resources, TNR programs can function as effective tools for reducing the ecological footprint of free-roaming cat populations.

## Figures and Tables

**Table 1:** Stable (δ^13^C, δ^15^N, and δ^34^S) isotope results for the baseline considered in this study.

Sample ID	Material	δ^13^C (‰)	δ^15^N (‰)	δ^34^S (‰)
4098	Rat bone	−21.9	9.1	6.0
4099	Wet food	−23.8	7.4	7.0
4100	Dry food	−19.3	3.3	3.9
4101	Dry food	−18.9	4.2	4.8
4102	Crow feather	−20.2	11.4	4.4
4103	Crow feather	−20.4	10.0	6.7
4104	Bird feather	−21.0	9.5	3.4
4105	Bird feather	−21.5	10.2	3.4

## Data Availability

Data is contained within the article or Supplementary Materials.

## Supplementary Materials

The following supporting information can be downloaded at: https://www.mdpi.com/article/10.3390/ani15213204/s1. File S1. List of all samples used in this study, including information on sex, estimated birthdate, sterilization date, colony, fur collection date, start feeding date, diet before and after TNR, and δ^13^C, δ^15^N, and δ^34^S isotope results. File S2. Laboratory report file containing the stable isotope data of cat fur samples. File S3. R code used for the statistical and isotopic analyses performed in this study. File S4. δ^13^C vs. δ^15^N and δ^15^N vs. δ^34^S scatterplots of the free-roaming cats analyzed in this study, by sterilization date. File S5. *p*-values from Wilcoxon rank-sum tests (with Holm correction) for pairwise comparisons of isotopic values (δ^13^C, δ^15^N, δ^34^S) among cat colonies and groups. File S6. csv files containing the datasets used for the R analyses.

## Abbreviations

The following abbreviations are used in this manuscript:

TNR	Trap-neuter-return
TKS	TinyKittens Society
SIA	Stable isotope analysis
TDF	Trophic discrimination factor
VPDB	Vienna Pee Dee Belemnite
AIR	Ambient Inhalable Reservoir
V-CDT	Vienna Canyon Diablo Troilite
