# Supplementary material for: Evaluating the Effects of Managed Free-Roaming Cat Populations on Prey Through Stable Isotope Analysis: A Pilot Study from British Columbia, Canada
Source: Animals (Basel). 2025 Nov 4;15(21):3204. doi: 10.3390/ani15213204 (PMC12606756; doi:10.3390/ani15213204)
Supplement: Supplementary file 1 [file animals-15-03204-s001.zip › Supplementary Material S3.pdf]

R code for statistical analyses
Browse[1]> sessionInfo()
R version 4.3.1 (2023-06-16)

>set.seed(1234)

library(dplyr)
library(tidyr)
library(coin)
library(broom)
library(rcompanion)
library(readr)
library(tidyverse)
library(cluster)

#### GROUP 1
G1_stats <- read.csv("cg_G1_STATS.csv", header = TRUE)

> ##### SEX
> kruskal.test(d13C ~ sex, data =
G1_stats)   Kruskal-Wallis rank
sum test data: d13C by sex
Kruskal-Wallis chi-squared = 0.36759, df = 2, p-value = 0.8321

> kruskal.test(d15N ~ sex, data =
G1_stats)   Kruskal-Wallis rank
sum test data: d15N by sex
Kruskal-Wallis chi-squared = 27.347, df = 2, p-value = 1.153e-06

> kruskal.test(d34S ~ sex, data = G1_stats)
    Kruskal-Wallis rank sum test
data: d34S by sex
Kruskal-Wallis chi-squared = 24.21, df = 2, p-value = 5.531e-06

> pairwise.wilcox.test(G1_stats$d13C, G1_stats$sex, p.adjust.method =
"holm")   Pairwise comparisons using Wilcoxon rank sum test with
continuity correction data: G1_stats$d13C and G1_stats$sex
  cg
Female
Female 1   -
Male   1 1
P value adjustment method: holm

> pairwise.wilcox.test(G1_stats$d15N, G1_stats$sex, p.adjust.method =
"holm")   Pairwise comparisons using Wilcoxon rank sum test with
continuity correction data: G1_stats$d15N and G1_stats$sex

cg Female
Female 3.5e-06 -
Male 2.4e-06 0.82

Pairwise comparisons using Wilcoxon rank sum exact test
data: G1_stats$d34S and G1_stats$sex
cg Female
Female 1.7e-07 -
Male 2.4e-05 0.23

SUBCOLONY
> kruskal.test(d13C ~ subcolony, data =
G1_stats) Kruskal-Wallis rank sum test
data: d13C by subcolony
Kruskal-Wallis chi-squared = 11.49, df = 3, p-value = 0.009351

> kruskal.test(d15N ~ subcolony, data =
G1_stats) Kruskal-Wallis rank sum test
data: d15N by subcolony
Kruskal-Wallis chi-squared = 50.651, df = 3, p-value = 5.805e-11

> kruskal.test(d34S ~ subcolony, data =
G1_stats) Kruskal-Wallis rank sum test
data: d34S by subcolony
Kruskal-Wallis chi-squared = 47.628, df = 3, p-value = 2.556e-10

> pairwise.wilcox.test(G1_stats$d13C, G1_stats$subcolony, p.adjust.method
= "holm") Pairwise comparisons using Wilcoxon rank sum test with
continuity correction data: G1_stats$d13C and G1_stats$subcolony
cg Lower Middle
Lower 1.000 - -
Middle 1.000 1.000 -
Upper 0.918 0.002
0.846

P value adjustment method: holm

> pairwise.wilcox.test(G1_stats$d15N, G1_stats$subcolony, p.adjust.method
= "holm") Pairwise comparisons using Wilcoxon rank sum test with
continuity correction data: G1_stats$d15N and G1_stats$subcolony
cg Lower Middle
Lower 6.8e-06 - -
Middle 0.00019 0.92708 -
Upper 8.2e-06 3.0e-07 0.01196
P value adjustment method: holm

> pairwise.wilcox.test(G1_stats$d34S, G1_stats$subcolony, p.adjust.method = "holm")   Pairwise comparisons using Wilcoxon rank sum test with continuity correction data: G1_stats$d34S and G1_stats$subcolony
       cg   Lower   Middle
Lower 9.0e-06 -   -
Middle 0.00025 0.72808 -
Upper 0.00031 1.4e-06
0.00031
P value adjustment method: holm

STERILIZATION
> STER <- read.csv("sterilization.csv", header = TRUE)
> STER$sterilized <- factor(STER$sterilized)

> kruskal.test(d13C ~ sterilized, data = STER)
Kruskal-Wallis rank sum test
data: d13C by sterilized
Kruskal-Wallis chi-squared = 16.022, df = 5, p-value = 0.006783

> kruskal.test(d15N ~ sterilized, data = STER)
Kruskal-Wallis rank sum test
data: d15N by sterilized
Kruskal-Wallis chi-squared = 23.135, df = 5, p-value = 0.0003181

> kruskal.test(d34S ~ sterilized, data = STER)
Kruskal-Wallis rank sum test
data: d34S by sterilized
Kruskal-Wallis chi-squared = 21.122, df = 4, p-value = 0.0002995

> pairwise.wilcox.test(STER$d13C, STER$sterilized, p.adjust.method = "holm")   Pairwise comparisons using Wilcoxon rank sum test with continuity correction data: STER$d13C and STER$sterilized

      01_23 02_23 03_23 04_23 05_23
02_23 0.059 -   -   -   -   03_23
1.000 1.000 -   -   -   04_23
0.026 1.000 1.000 -   -   05_23
1.000 1.000 1.000 1.000 -
06_23 1.000 1.000 1.000 1.000
1.000
P value adjustment method: holm

> pairwise.wilcox.test(STER$d15N, STER$sterilized, p.adjust.method = "holm")   Pairwise comparisons using Wilcoxon rank sum test with continuity correction data: STER$d15N and STER$sterilized
      01_23 02_23 03_23 04_23 05_23

02_23 0.003 - - - - 03_23
0.025 1.000 - - - 04_23
0.049 1.000 1.000 - - 05_23
1.000 1.000 1.000 1.000 -
06_23 1.000 1.000 1.000 1.000
1.000
P value adjustment method: holm

> STER_noNA <- subset(STER, !is.na(d34S))
> pairwise.wilcox.test(STER_noNA$d34S, STER_noNA$sterilized,
p.adjust.method = "holm") Pairwise comparisons using Wilcoxon rank sum
test with continuity correction data: STER_noNA$d34S and
STER_noNA$sterilized

01_23 02_23 03_23 04_23
02_23 0.0010 - - -
03_23 0.0027 1.0000 - -
04_23 0.0776 1.0000 1.0000 -
06_23 0.8602 1.0000 1.0000
1.0000
P value adjustment method: holm

#### GROUP 2
> G2_stats <- read.csv("G2.csv", header = TRUE)
> View(G2_stats)

#### GROUP
> kruskal.test(d13C ~ group, data = G2_stats)

Kruskal-Wallis rank sum test
data: d13C by group
Kruskal-Wallis chi-squared = 68.624, df = 3, p-value = 8.413e-15

> kruskal.test(d15N ~ group, data =
G2_stats) Kruskal-Wallis rank sum
test data: d15N by group
Kruskal-Wallis chi-squared = 93.014, df = 3, p-value < 2.2e-16

> kruskal.test(d34S ~ group, data =
G2_stats) Kruskal-Wallis rank sum
test data: d34S by group
Kruskal-Wallis chi-squared = 71.108, df = 3, p-value = 2.471e-15

> pairwise.wilcox.test(G2_stats$d13C, G2_stats$group, p.adjust.method =
"holm") Pairwise comparisons using Wilcoxon rank sum test with
continuity correction data: G2_stats$d13C and G2_stats$group cg

Group 1 Group 2 Group 1 0.5672 - - Group 2 0.0017 6.5e-11 - Run 2
0.0017 3.4e-09 0.4039
P value adjustment method: holm

> pairwise.wilcox.test(G2_stats$d15N, G2_stats$group, p.adjust.method = "holm") Pairwise comparisons using Wilcoxon rank sum test with continuity correction data: G2_stats$d15N and G2_stats$group cg Group 1 Group 2 Group 1 7.2e-07 - - Group 2 0.92 7.0e-15 - Run 2 0.92 8.4e-10 0.92
P value adjustment method: holm

> pairwise.wilcox.test(G2_stats$d34S, G2_stats$group, p.adjust.method = "holm") Pairwise comparisons using Wilcoxon rank sum test with continuity correction data: G2_stats$d34S and G2_stats$group cg
Group 1 Group 2 Group 1 6.8e-06 - -
Group 2 0.00075 1.3e-10 -
Run 2 0.00075 4.3e-06
0.00396
P value adjustment method: holm

Group 1 vs Run 2

```
df <- read_csv("G1_R2_17.csv", show_col_types = FALSE)
Browse[1]> print(unique(df$group))
[1] "Group 1" "Run 2"
Browse[1]> paired <- df %>%
+   filter(group %in% c("Group 1","Run 2")) %>%
+   distinct(name, group, .keep_all = TRUE) %>%
+   group_by(name) %>%
+   filter(n() == 2) %>%
+   ungroup()
Browse[1]> message("Paired cats found: ", dplyr::n_distinct(paired$name))
Paired cats found: 17
Browse[1]> paired_wilcoxon_simple <- function(dat, var){
+   wide <- dat %>%
+     select(name, group, val = all_of(var)) %>%
+     pivot_wider(names_from = group, values_from = val) %>%
+     filter(!is.na(`Group 1`) & !is.na(`Run 2`))
+
+   x_pre  <- wide$`Group 1`
+   x_post <- wide$`Run 2`
+
+   ### Paired Wilcoxon
+   wt <- suppressWarnings(
+     wilcox.test(x_post, x_pre, paired = TRUE, exact = FALSE,
+           conf.int = TRUE, conf.level = 0.95)
+   )
+   V  <- unname(wt$statistic)
```

```
+   p  <- wt$p.value
+   HL <- unname(wt$estimate)          # Hodges–Lehmann (post - pre)
+   CI <- wt$conf.int
+
+   ### Effect size r (from z-approx of p), sign from median difference
+   diffs <- x_post - x_pre
+   n     <- length(diffs)
+   z_abs <- qnorm(p/2, lower.tail = FALSE)       # two-tailed z magnitude
+   z     <- z_abs * sign(median(diffs, na.rm = TRUE))
+   r     <- z / sqrt(n)
+
+   ### Matched-pairs rank-biserial correlation (ignoring exact zero diffs)
+   diffs_nz <- diffs[diffs != 0 & !is.na(diffs)]
+   n_eff    <- length(diffs_nz)
+   if (n_eff > 0) {
+     ranks  <- rank(abs(diffs_nz))
+     Rplus  <- sum(ranks[diffs_nz > 0])
+     Rminus <- sum(ranks[diffs_nz < 0])
+     Tsum   <- n_eff * (n_eff + 1) / 2
+     r_rb   <- (Rplus - Rminus) / Tsum
+   } else {
+     r_rb <- NA_real_
+   }
+
+   tibble(
+     variable      = var,
+     n_pairs       = n,
+     V             = as.numeric(V),
+     p_value       = p,
+     HL_shift      = HL,
+     HL_CI_low     = CI[1],
+     HL_CI_high    = CI[2],
+     r_effect      = r,
+     rank_biserial = r_rb
+   )
+ }

Browse[1]> results <- bind_rows(
+   paired_wilcoxon_simple(paired, "d13C"),
+   paired_wilcoxon_simple(paired, "d15N"),
+   paired_wilcoxon_simple(paired, "d34S")
+ )

Browse[1]>
print(results) # A tibble:
3 × 9
```

```
  variable n_pairs     V  p_value HL_shift HL_CI_low HL_CI_high r_effect
rank_biserial
  <chr>      <int> <dbl>    <dbl>    <dbl>     <dbl>      <dbl>    <dbl>         <dbl>
1 d13C        17   152 0.000385     1.45      1.26       1.60    0.861         0.987
2 d15N        17     0 0.000321    -3.96     -4.47      -3.28   -0.873        -1
3 d34S        17     2 0.000460    -8.85     -9.66      -5.43   -0.850        -0.974
```

R code for SIBER

```
> library(SIBER)
> cats <- read.csv("CN.csv") #NS.csv for N-S
> cats$group <- factor(cats$group,
+                      levels = c(1, 2, 3, 4),
+                      labels = c("Indoor", "Group 1", "Group 2", "Run 2"))
> split_dat <- split(cats, cats$group)

> siberEllArea <- function(Sigma) {
+   pi * sqrt(det(Sigma))
+ }

> SEA_values <- sapply(split_dat, function(df) {
+   Sigma <- cov(df[, c("iso1", "iso2")])
+   siberEllArea(Sigma)
+ })
> SEA_df <- data.frame(
+   Group = names(SEA_values),
+   SEA = SEA_values
+ )
> print(SEA_df) #for C-N
          Group       SEA
Indoor   Indoor 1.7579165
Group 1 Group 1 3.6244109
Group 2 Group 2 1.3251747
Run 2     Run 2 0.1734777

> print(SEA_df) #for N-S
          Group       SEA
Indoor   Indoor 1.7656503
Group 1 Group 1 7.6235612
Group 2 Group 2 4.1004724
Run 2     Run 2 0.2997414

> library(ellipse)
> library(sp)
> library(sf)
> cols <- c("Indoor" = "#97ce30",
```

```
+         "Group 1" = "#d54344",
+         "Group 2" = "#9069d6",
+         "Run 2"   = "#4cb1df")
> plot(cats$iso1, cats$iso2,
+     pch = 16,
+     col = cols[cats$group],
+     xlab = expression(delta^13*C~"\u2030"),
+     ylab = expression(delta^15*N~"\u2030"),
+     asp = 1)
> split_dat <- split(cats, cats$group)
> ellipse_poly <- function(df, level = 0.4) {
+   mu <- colMeans(df[, c("iso1", "iso2")])
+   Sigma <- cov(df[, c("iso1", "iso2")])
+   e <- ellipse(Sigma, centre = mu, level = level, npoints = 200)
+   Polygon(rbind(e, e[1, ]))
+ }
> polygons <- lapply(split_dat, ellipse_poly)
> for (g in names(polygons)) {
+   lines(polygons[[g]]@coords, col = cols[g], lwd = 2)
+ }
> legend("topright",
+       legend = names(cols),
+       col = cols,
+       lwd = 2, pch = 16,
+       bty = "n")
> ellipses_closed <- lapply(polygons, function(p) {
+   Polygon(rbind(p@coords, p@coords[1, ]))
+ })
> polygons_sp <- mapply(
+   function(poly, name) SpatialPolygons(list(Polygons(list(poly), name))),
+   ellipses_closed, names(ellipses_closed),
+   SIMPLIFY = FALSE
+ )

> sf_polys <- lapply(split_dat, function(df) {
+   mu <- colMeans(df[, c("iso1","iso2")])
+   Sigma <- cov(df[, c("iso1","iso2")])
+   e <- ellipse(Sigma, centre = mu, level = 0.4, npoints = 200)
+   e <- rbind(e, e[1, ])  # close polygon
+   st_polygon(list(as.matrix(e))) |> st_sfc()
+ })
> names(sf_polys) <- names(split_dat)
> overlap_percent_sf <- function(p1, p2) {
+   inter <- suppressWarnings(st_intersection(p1, p2))
+   if (length(inter) == 0) return(0)
+   overlap_area <- st_area(inter)
```

```
+   min_area <- min(st_area(p1), st_area(p2))
+   as.numeric(overlap_area / min_area * 100)
+ }
> n <- length(sf_polys)
> overlap_mat <- matrix(0, n, n, dimnames = list(names(sf_polys),
names(sf_polys)))
> for (i in 1:(n - 1)) {
+   for (j in (i + 1):n) {
+     overlap_mat[i, j] <- overlap_percent_sf(sf_polys[[i]], sf_polys[[j]])
+     overlap_mat[j, i] <- overlap_mat[i, j]
+   }
+ }
> round(overlap_mat, 2) #for C-N
        Indoor Group 1 Group 2 Run 2
Indoor    0.00       0    6.61     0
Group 1   0.00       0    0.00     0
Group 2   6.61       0    0.00   100
Run 2     0.00       0  100.00     0

> round(overlap_mat, 2) #for N-S
        Indoor Group 1 Group 2 Run 2
Indoor    0.00       0   63.83     0
Group 1   0.00       0    0.00     0
Group 2  63.83       0    0.00   100
Run 2     0.00       0  100.00     0
```
