# Supplementary material for: Evaluating the Effects of Managed Free-Roaming Cat Populations on Prey Through Stable Isotope Analysis: A Pilot Study from British Columbia, Canada
Source: Animals (Basel). 2025 Nov 4;15(21):3204. doi: 10.3390/ani15213204 (PMC12606756; doi:10.3390/ani15213204)
Supplement: Supplementary file 1 [file animals-15-03204-s001.zip › Supplementary Material S4.pdf]

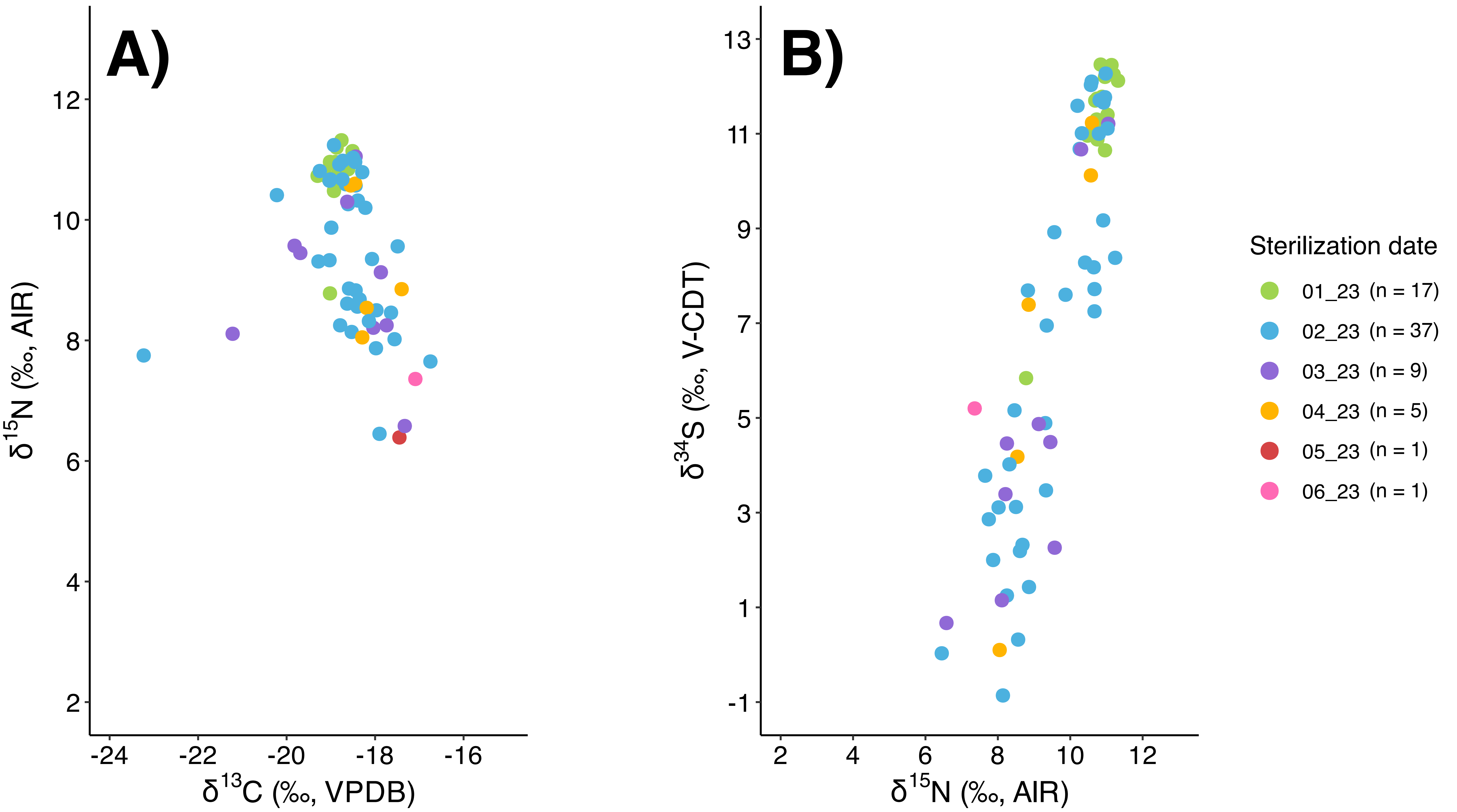

Scatterplots of: A) δ^{13}C vs. δ^{15}N and B) δ^{15}N vs. δ^{34}S values of the free-roaming cats from Group 1 analyzed in this study, divided by date of sterilization (in the format MM_YY).

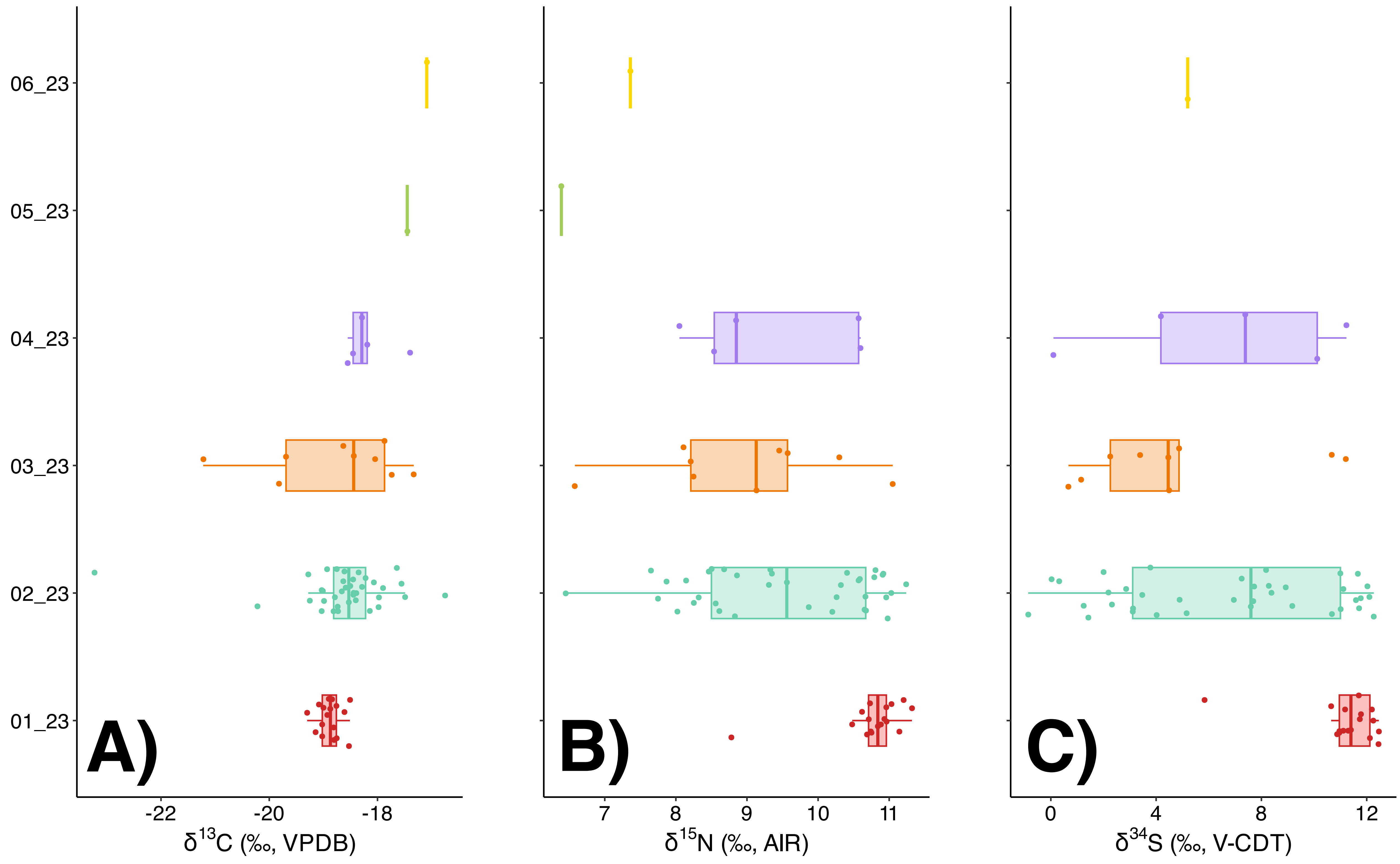

Box- and jitter-plots of: A) δ^{13}C, (B) δ^{15}N, and C) δ^{34}S values of the free-roaming cats from Group 1 analyzed in this study, divided by date of sterilization (in the format MM_YY).
